# Forefoot disease activity in rheumatoid arthritis patients in remission: results of a cohort study

**DOI:** 10.1186/ar2901

**Published:** 2010-01-07

**Authors:** Marike van der Leeden, Martijn PM Steultjens, Dirkjan van Schaardenburg, Joost Dekker

**Affiliations:** 1 Department of Rehabilitation Medicine and Psychology, Jan van Breemen Institute, Jan van Breemenstraat 2, 1056 AB Amsterdam, The Netherlands; 2 Department of Rehabilitation Medicine, VU University Medical Center EMGO Institute, De Boelelaan 1118, 1081 HZ Amsterdam, The Netherlands; 3 Department of Rheumatology, Jan van Breemen Institute, Jan van Breemenstraat 2, 1056 AB, Amsterdam, The Netherlands; 4 Department of Rheumatology, VU University Medical Center, De Boelelaan 1118, 1081 HZ Amsterdam, The Netherlands

## Abstract

**Introduction:**

The aim of our study was to investigate the presence of disease activity in the metatarsophalangeal (MTP) joints of the forefoot in rheumatoid arthritis (RA) patients in remission according to the Disease Activity Score based on 28 joints (DAS28) remission criterion.

**Methods:**

A total of 848 patients with recent-onset RA were included from 1995 through 2007. The DAS28 and pain and swelling of the MTP joints were assessed annually. The data were analyzed using descriptive techniques.

**Results:**

On average, 35% of the patients fulfilled the remission criterion of DAS28 <2.6 during the first eight years of RA. On average, 29% of these patients had at least one painful MTP joint and, on average, 31% had at least one swollen MTP joint during follow-up. Forty percent, on average, had at least one involved MTP joint (pain and/or swelling).

**Conclusions:**

Painful and/or swollen MTP joints were detected in a substantial proportion of patients classified as being in remission. Therefore, examination of the foot joints - irrespective of the patient's state of remission - seems indicated in order to provide optimal foot care.

## Introduction

Forefoot disease activity appears to be frequent in rheumatoid arthritis (RA). In a cohort of patients with recent-onset RA and a maximum of eight years follow up, prevalence rates for pain and swelling of the metatarsophalangeal (MTP) joints were initially high and then stabilized at around 40% during the eight-year course of RA [1]. Forefoot disease activity can lead to joint damage, pain and disability in weight bearing activities.

The medical treatment policy is often based on the disease activity score with a 28 joint count (DAS28)[2]. The feet are omitted in this score, as they are less easily accessible for clinical examination in daily practice than the hands. The DAS28 can be used to define clinical remission of the disease. Values of the DAS28 below 2.6 are reported to correspond with being in clinical remission [3]. Whether forefoot disease activity is present in patients who are in remission according to the DAS28 is unknown. Therefore, the aim of the present study is to investigate forefoot disease activity in RA patients in remission.

## Materials and methods

### Study design

Since 1995, patients age >18 years with recent-onset arthritis (peripheral arthritis of >2 joints and symptom duration less than three years) have been included in the early arthritis cohort (EAC) [4]of the Jan van Breemen Institute (a large rheumatology clinic in Amsterdam, The Netherlands). In the EAC, the patients' disease activity, joint damage, and functional capacity have been assessed at different time points. The local ethics committee (Slotervaart Hospital and Jan van Breemen Institute, Amsterdam, The Netherlands) approved the study protocol. All patients gave written informed consent to be included in the study. Drug treatment decisions were made by the rheumatologists according to clinical practice standards.

For the present study, all patients included between 1995 and May 2007 and fulfilling the 1987 American College of Rheumatology (ACR; formerly the American Rheumatism Association) criteria for RA [5] at baseline and/or at one year after inclusion were selected. Data from annual assessments were used, with a maximum of eight years of follow up.

### Measures

#### Disease activity

Global disease activity with a 28-joint count (DAS28) was determined at every annual measurement point [2]. Beside the 28-joint count, pain and swelling of the MTP joints were assessed annually. The DAS28 and the number of painful and/or swollen MTP joints per measurement point were used for the analysis.

#### Baseline patient characteristics

Demographic data, duration of symptoms, IgM rheumatoid factor (RF), joint damage of the hands and feet scored with the Sharp/van der Heijde method (in a subgroup of patients with a follow up ≥ 2 years), and functional capacity measured with the Health Assessment Questionnaire (HAQ) were recorded at baseline.

### Statistical analysis

Means and medians were calculated for the characteristics of the patients at baseline. Additionally, the percentage of patients in remission (DAS28 <2.6) was calculated at every annual measurement point from baseline to a maximum of eight years of follow up. From these patients in remission the percentage of patients who had 0, 1, 2 to 4 or 5 to 10 painful and/or swollen MTP joints was calculated for each year of follow up and visualised in graphs.

All analyses were performed using SPSS, version 15.0 (SPSS, Chicago, IL).

## Results

A total of 848 (52%) patients fulfilled the ACR criteria for RA within the first year and were included in our study. These 848 patients had at least a baseline measurement. The duration of follow-up varied between patients, as patients have been included from 1995 through May 2007. A total of 682 patients had a follow-up of one year, 341 patients had a follow-up of four years and 121 patients had a full follow-up of eight years. In our earlier report on the same cohort, we have analyzed the drop-out from the cohort. The most common reasons for patients to drop out were no time (n = 81), moving to another area (n = 46), and remission of the disease (n = 37). Selection bias was concluded to be minimal as a result of drop-out [1].

The baseline characteristics of the patients are shown in Table 1.

**Table 1:** Patient characteristics at baseline (N = 848)

Characteristic	Value
**Female, %**	69
**Age, mean (SD) years**	55.2 (14.2)
**Duration of symptoms, median (IQR)**	0.0 (0.0 to 1.0)
**Rheumatoid factor positive, %**	51.3
**DAS28, mean (SD)**	5.2 (1.2)
**SHS total, median (IQR)/mean (SD)**	0.0 (0.0 to 3.0)/4.1(12.2)

**HAQ DI total score, median (IQR)**	1.1 (0.6 to 1.9)

**Figure 1:**
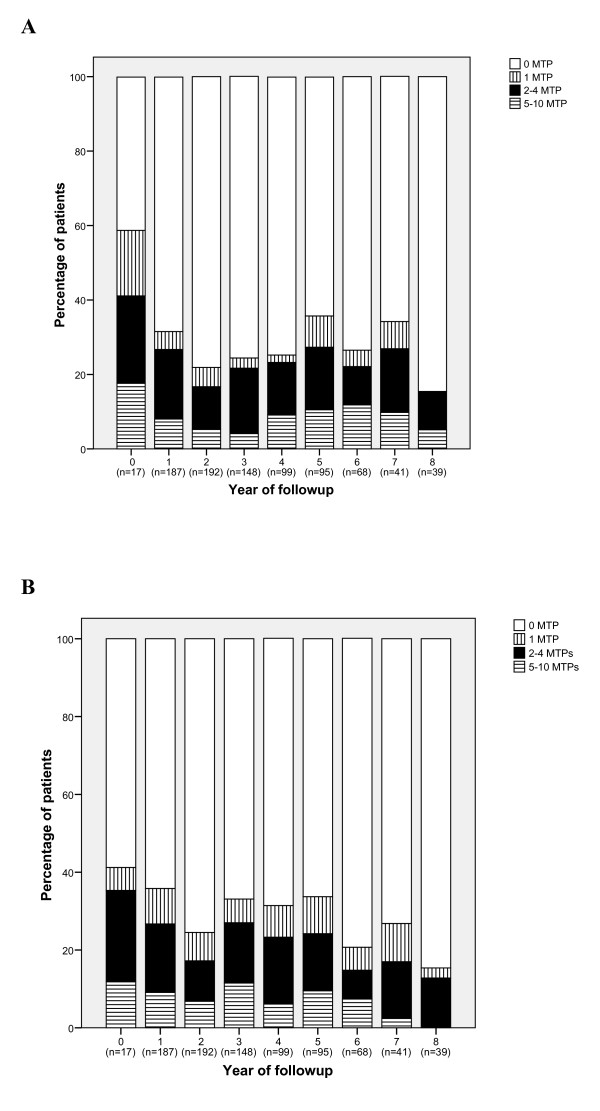
Percentage of patients in remission with 0, 1, 2 to 4 and 5 to 10 painful (a) or swollen (b) MTP joints

DAS28 = Disease Activity Score in 28 joints; HAQ DI = Health Assessment Questionnaire disability index; IQR = Interquartile range; SHS total = total Sharp/van der Heijde score for a subgroup of patients with a follow up ≥ 2 years;

On average, 35% (range 28% to 40%) of the patients fulfilled the remission criterion of DAS28 <2.6 during follow-up (that is, from Year 1 to Year 8; at baseline only 2% of the patients were in remission). Figure 1 shows which percentage of the patients in remission had 0, 1, 2 to 4, and 5 to 10 painful (Figure 1a) and swollen MTP (Figure 1b) joints per measurement point. On average, 29% of these patients had at least one painful MTP joint. On average, 31% had at least one swollen MTP joint during follow-up. Forty percent, on average, had at least one involved MTP joint (pain and/or swelling). The percentage of patients in remission with at least five involved MTP joints was on average 7% and 9% for pain and swelling, respectively.

## Discussion

The results of the present study showed that a substantial proportion of RA patients, considered to be in remission according to the DAS28 criterion, had disease activity in the forefoot joints. About 40% of the patients in remission had at least one painful and/or swollen MTP joint during the first eight years of RA. This suggests that the DAS28 remission criterion for RA neglects patients with active forefoot involvement.

The DAS28 cut-off point for remission of 2.6 has been discussed in several studies. The original DAS, including a 44-joint count, has a cut-off point of 1.6 for remission [6]. Landewé *et al *(2006) found that the DAS remission criterion of the original DAS is more conservative than the DAS28 remission criterion [7]. This discrepancy can be accounted for by the assessment of disease activity in joints, such as ankles and feet that are excluded from the DAS28. The study concluded that the DAS28 cut-off point of 2.6 for RA remission has insufficient construct validity and should therefore be used with caution in clinical practice and trials. Mäkinen *et al *(2005) found that a substantial proportion of patients, who had a DAS28 score below the 2.6 cut-off point, had tender and/or swollen joints and therefore concluded that the DAS28 may not be an appropriate tool for assessment of remission in RA [8]. However, Kapral et al (2007) compared a 28-joint count with a 32-joint count (including ankles and MTP joints) in composite indices and concluded that reduced joint counts (without ankles and MTP joints) in composite indices are appropriate and valid tools for disease activity assessment [9]. Furthermore, a study of van der Heijde *et al *(2005) showed no differences between the DAS44 and DAS28 in assessing remission [10]. As a consequence of these findings and given that the DAS28 is easier to use than the original DAS, the DAS28 remission criterion remains frequently used to assess remission in clinical practice and clinical trials.

Our results showed that a substantial proportion of patients classified as being in remission according to the DAS28 criterion had disease activity in the MTP joints. Because undetected disease activity can result in non optimal foot care, we recommend examining the foot joints when using the DAS28 remission criterion in clinical practice. Optimal foot care for patients with RA can be achieved using both medical and conservative modalities.

A limitation of our study is that we report on data of disease activity in MTP joints only. Although MTP joints are most commonly affected in RA, further research should include disease activity in other foot joints beside the MTP joints.

In our earlier report on the same cohort, we investigated the eight-year course of joint damage in the MTP joints in RA patients. It was shown that the forefoot erosion score was ≥ 1 in 19% of the patients at baseline, and the prevalence of forefoot erosion increased to approximately 60% after eight years, during which the mean forefoot erosion score increased from 1.3 to 7.9 [1]. The present study focuses on disease activity in MTP joints, rather than on joint damage. A suggestion for future research is to evaluate joint damage in the feet of patients with persistent remission according to the DAS28.

## Conclusions

Of the patients in remission, 40%, on average, had disease activity (pain and/or swelling) in at least one MTP joint during the first eight years of RA. On average 29% of these patients had at least one painful MTP joint, and on average 31% had at least one swollen MTP joint during follow-up.

Given that painful and/or swollen MTP joints were detected in patients classified as being in remission according to the DAS28, examination of the foot joints - irrespective of the patient's state of remission - seems indicated in order to provide optimal foot care.

## Abbreviations

ACR: American College of Rheumatology; DAS28: Disease Activity Score based on 28 joints; EAC: Early Arthritis Cohort; HAQ: Health Assessment Questionnaire; MTP joints: metatarsophalangeal joints; RA: rheumatoid arthritis; RF: rheumatoid factor; SHS: Sharp/van der Heijde score.

## Competing interests

The authors declare that they have no competing interests.

## Authors' contributions

ML collected additional data in the Early Arthritis Cohort, performed the statistical analysis and wrote the manuscript. MS helped with the statistical analysis, participated in the design of the study and helped to draft the manuscript. DS collected additional data in the Early Arthritis Cohort, participated in its design and helped to draft the manuscript. JD participated in its design and coordination and helped to draft the manuscript. All authors read and approved the final manuscript.
